# High SNRPA1 expression leads to poor prognosis in patients with lung adenocarcinoma

**DOI:** 10.1111/crj.13647

**Published:** 2023-06-05

**Authors:** Juan Juan Yang, Yu Jia Yang, Yi Lu Gu, Li Tong, Yi Fei Liu, Jian Guo Zhang

**Affiliations:** ^1^ Research Center of Clinical Medicine Affiliated Hospital of Nantong University Nantong Jiangsu China; ^2^ Department of Pathology Affiliated Hospital and Medical School of Nantong University Nantong Jiangsu China

**Keywords:** immune therapy, lung adenocarcinoma, prognosis marker, RNA‐binding proteins, SNRPA1

## Abstract

**Objective:**

*SNRPA1*, a subunit of spliceosome complex, has been implicated in diverse cancers, while its biological effect in LUAD remains elusive. Therefore, we sought to decipher the relationship between SNRPA1 expression and the prognosis of patients with LUAD and reveal the underlying molecular mechanism.

**Materials and methods:**

Based on the clinical data from TCGA databases, the multivariate Cox model was constructed to screen the prognostic value of SNRPA1. qRT‐PCR and immunohistochemical staining were used to examine *SNRPA1* mRNA and protein expression in LUAD. The effect of *SNRPA1* on LUAD cell proliferation, migration, and epithelial mesenchymal transformation were examined using colony formation assays, wound healing, and western blot assays, respectively. Finally, the influence of SNRPA1 on LUAD immune microenvironment were validated from the Tumor Immune Estimation Resource database.

**Results:**

*SNRPA1* was significantly upregulated in both LUAD tissues and cell lines, and highly expressed SNRPA1 contributed to poor prognosis of LUAD patients. In vitro, *SNRPA1* knockdown inhibited the proliferation and migration, as well as delayed the EMT differentiation of LUAD cells. Lastly, SNRPA1 was found to be positively associated with immune infiltration and some immune‐check‐point markers.

**Conclusions:**

Our findings indicate that SNRPA1 may be a new biomarker for prognostic prediction and a potential therapeutic target in the treatment of LUAD.

## Graphical abstract

We analyzed the clinical correlation of prognostic RNA‐binding proteins (SNRPA1) with the expression profiles. Moreover, we verified the function of SNRPA1 in additional clinical samples and in vitro experiments. Collectively, we evidenced the prognostic significance of *SNRPA1* in LUAD and indicated its role correlated with immune filtration, which may be useful in predicting survival and individualized treatment in LUAD.

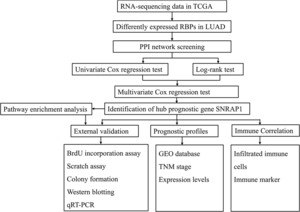

## 1 INTRODUCTION

The diagnosed new cases for lung cancer increases with years,^1^ and an estimated 40% of lung cancers belongs to lung adenocarcinoma.^2^ Despite recent development in diagnostic and treatment, the prognosis of LUAD remains unfavorable due to a lack of early diagnoses.^3^ Therefore, it is urgent to identify some new prognostic markers to improve the prognosis of LUAD.

RNA‐binding proteins (RBPs) are a large group of proteins that facilitate some biological process of cellular RNAs.^4^ The dysregulation of RBPs leads to alteration in RNA metabolism, in turn affecting cell growth and invasion.^5^, ^6^ Accumulating evidence indicates that RBPs dysregulation contributes to the alteration of oncogenes or tumor suppressor genes.^7^ Small nuclear ribonucleoprotein polypeptide A (*SNRPA1*), a spliceosome component responsible for the splicing reactions of precursor messenger RNAs (pre‐mRNAs), is upregulated in various cancers.^8^, ^9^ Chen et al.^10^ revealed that nuclear *SNRPA1* can enhance the ubiquitination degradation of p53 through their interaction, and result in the proliferation of colorectal cancer. In breast cancer,^11^ *SNRPA1* interacts with gene enhancers to promote cassette exon inclusion, and leads to the formation of metastatic cancer colonization and cell invasion. Furthermore, Jiang et. al^9^ found that SNRPA1 expression was positively correlated with the clinical stage and overall survival of cell renal cell carcinoma cancer patients. They discovered that elevated SNRPA1 contributed to cell invasion and cancer metastasis; blockage of *SNRPA1* is a promising synergistic antitumor strategy for sunitinib sensitivity and anti‐PD‐1 immunotherapy.

Although a comprehensive comparison of gene expression profiling between lung adenocarcinoma tissues and normal samples identified eight immune‐related genes RBPs for prognostic and efficacious prediction of LUAD patients,^12^ SNRPA1 was not included. In this study, we found four RBPs (*GAPDH*, *IGF2BP1*, *PABPC1L*, and *SNRPA1*) highly expressed in LUAD through comprehensive biomarker discovery and further high content screening. And our results indicated that knock down of *SNRPA1* inhibited cell proliferation and migration of LUAD cells. Further function enrichment analyses and validation revealed that SNRPA1 played its role via promoting epithelial–mesenchymal transition (EMT) differentiation. In summary, those results displayed the novel role of SNRPA1 in LUAD, and it may become a new prognostic marker and therapeutic target for the treatment of LUAD.

## 2 MATERIALS AND METHODS

### 2.1 Data collection

RNA sequencing data of LUAD, including 497 LUAD samples and 54 normal controls, were downloaded from TCGA database. The mRNA expression profiles of 1495 RBPs were collected and analyzed using “Limma” package in R software, then differentially expressed genes (DEGs) with thresholds of |log2FC| > 1.0 and FDR value <0.05 were selected for further analysis.

### 2.2 Function enrichment analysis and PPI networks construction

KEGG pathway enrichment analyses were performed to elucidate the biological function of RBPs, and the results were visualized using R package “GOplot.” The interactions between differently expressed RBPs were analyzed based on STRING database (http://string-db.org). After disregarding disconnected nodes and screening using an interaction score ˃0.4, selected RBPs were further visualized by constructing the protein–protein interaction (PPI) network using Cytoscape software (Version 3.8.2). RBPs with degrees ≥10 were identified as hub RBPs using the cytoHubba plug‐in.

### 2.3 Prognostic analysis and multiple databases validation

Hub RBPs with *p* values <0.05 were selected for multivariate Cox model construction. Through the stepwise regression, prognosis‐related RBPs were identified and further used to assess the prognosis value based on Kaplan–Meier survival curves construction. Data from TCGA and another three datasets from the GEO (GSE68465, GSE31210, GSE72094) were downloaded and used to verify the expression profiles of prognostic RBPs.

### 2.4 RNA extraction and qRT‐PCR

Total of 16 pairs LUAD cancer tissues and adjacent normal controls were recruited from 2021 to 2022 at Affiliated Hospital of Nantong University. The study was approved by the Medical Ethics Committee of Affiliated Hospital of Nantong University (Nantong, China). The written informed consent was obtained from each patient prior to this study.

Total RNA from patient tissues and cells were isolated using TRIZol (Invitrogen, Carlsbad, CA, USA) reagent and reversed using the HiScript RT SuperMix for RT‐PCR Kit. HiScript® One Step qRT‐PCR SYBR® Green Kit (Vazyme, Nanjing, China) was employed to identify the levels of the genes. The mRNA levels were calculated using 2^−△△Ct^ method and normalized to β‐actin. The sequence of primers was shown in Table 1.

**TABLE 1:** Differentially expressed RBPs in LUAD and normal samples.

Gene	conMean	treatMean	logFC	*p* value	FDR
SMAD6	11.12644	1.53895	−2.85397	2.63E−29	2.56E−27
NOVA2	2.821055	0.488198	−2.5307	5.14E−32	4.62E−29
AFF3	3.588328	0.624269	−2.52307	2.71E−29	2.56E−27
RBMY1J	0.00425	0.000743	−2.51588	0.00019	0.000309
PIH1D3	1.833918	0.342235	−2.42187	5.99E−12	2.21E−11
NXF2B	0.003959	0.000862	−2.1993	7.90E−06	1.47E−05
NXF3	1.701813	0.396013	−2.10345	4.26E−25	1.23E−23
RNASE11	0.000272	6.74E−05	−2.01304	0.000533	0.000826
ADARB1	9.372388	2.374151	−1.981	6.12E−30	9.00E−28
APOBEC4	1.565599	0.398357	−1.97458	3.09E−12	1.16E−11
ZFP36	558.3072	147.0846	−1.92441	9.11E−19	7.88E−18
TLR8	7.344986	1.945637	−1.91652	2.32E−25	7.13E−24
KHDRBS2	3.836767	1.060479	−1.85517	2.50E−24	5.90E−23
NXF2	0.000413	0.000129	−1.6809	0.000272	0.000434
TRIM71	0.564801	0.180478	−1.64592	7.81E−23	1.38E−21
RBMS3	5.840585	1.892944	−1.62548	3.95E−28	2.38E−26
L1TD1	0.616886	0.206822	−1.57661	1.63E−25	5.40E−24
QKI	19.282	6.494763	−1.56991	2.28E−30	4.06E−28
SMAD9	5.197269	1.767163	−1.55632	1.10E−26	4.57E−25
SECISBP2L	29.1056	10.01893	−1.53857	1.97E−27	9.33E−26
ZNF106	17.58846	6.12024	−1.52297	2.29E−29	2.53E−27
RBMS2	23.61664	8.244995	−1.51821	1.26E−30	3.39E−28
ENDOU	0.256365	0.090268	−1.50591	2.25E−23	4.51E−22
PPARGC1B	1.258877	0.458642	−1.4567	7.37E−24	1.63E−22
CTIF	9.865604	3.610083	−1.45038	1.22E−28	9.54E−27
NCBP2L	0.163048	0.061806	−1.39948	3.31E−07	7.15E−07
CELF2	22.58284	8.563341	−1.39898	5.31E−27	2.27E−25
SMAD7	22.07721	8.379378	−1.39764	2.16E−28	1.51E−26
ADARB2	0.240755	0.093965	−1.35736	3.55E−20	3.82E−19
SIDT2	17.42747	6.813364	−1.35492	7.64E−30	1.01E−27
OASL	10.51245	4.142152	−1.34365	2.66E−19	2.41E−18
SAMHD1	71.73327	28.44953	−1.33424	1.36E−26	5.45E−25
RNASE13	0.036772	0.015004	−1.29327	8.70E−10	2.50E−09
ZCCHC24	14.87035	6.08817	−1.28836	8.23E−28	4.54E−26
NXF5	0.022727	0.009366	−1.27887	1.35E−06	2.71E−06
EIF4E3	6.805848	2.844386	−1.25866	3.16E−28	1.99E−26
N4BP1	20.99479	8.981747	−1.22496	4.98E−29	4.12E−27
LRRFIP1	29.76628	13.14045	−1.17966	1.15E−29	1.39E−27
IFIT1B	0.045365	0.02048	−1.14733	8.36E−07	1.73E−06
RBM24	0.544615	0.245958	−1.14683	7.55E−17	5.08E−16
IFIT2	15.17095	7.26027	−1.06322	4.61E−18	3.77E−17
ZC3H12A	20.68029	9.906213	−1.06185	0.009743	0.012964
SBDS	98.33751	47.20295	−1.05886	3.10E−28	1.99E−26
PABPC5	0.479903	0.234389	−1.03383	7.73E−16	4.70E−15
CPEB1	0.289939	0.142503	−1.02476	7.48E−21	8.84E−20
RNASE1	784.2045	388.8909	−1.01186	2.32E−16	1.51E−15
ZC3H12C	3.481137	1.737682	−1.00239	1.19E−21	1.73E−20
PUS1	2.080606	4.20157	1.013924	7.01E−21	8.36E−20
RNASEH2A	5.377812	10.97454	1.02907	4.83E−17	3.38E−16
URB1	2.683815	5.500462	1.035268	2.67E−21	3.50E−20
BRIX1	4.498972	9.230627	1.036833	4.27E−23	8.08E−22
DUS4L	0.950445	1.951313	1.03777	4.52E−23	8.42E−22
MEX3B	0.575434	1.185254	1.042473	6.11E−15	3.30E−14
BZW2	13.3873	27.62765	1.045247	1.75E−23	3.74E−22
INTS8	3.035138	6.269341	1.046552	4.07E−29	3.59E−27
MATR3	0.228208	0.471467	1.046806	3.02E−09	8.12E−09
DARS2	5.002109	10.34	1.047628	2.88E−24	6.68E−23
SPATS2	2.500258	5.217372	1.061246	6.39E−26	2.42E−24
CD3EAP	0.813838	1.716487	1.076646	1.06E−19	1.01E−18
KHDC1	0.429062	0.908597	1.082456	3.84E−16	2.44E−15
BARD1	1.182414	2.538305	1.10213	3.32E−21	4.31E−20
MRM1	2.311597	4.993896	1.111276	1.23E−22	2.15E−21
C2orf15	1.010244	2.189831	1.116116	4.28E−17	3.03E−16
MSI2	1.85843	4.104302	1.143053	1.68E−23	3.65E−22
PUS7	2.478527	5.514604	1.153774	2.60E−25	7.64E−24
JAKMIP1	0.307156	0.690524	1.16872	8.16E−07	1.69E−06
PABPC3	0.151966	0.343712	1.177453	7.84E−07	1.63E−06
METTL1	4.412032	10.10272	1.195229	5.78E−21	7.09E−20
MOV10L1	0.057174	0.130922	1.195273	0.006474	0.008819
DCAF13	2.821911	6.477291	1.198718	2.91E−26	1.13E−24
TDRKH	2.73895	6.646374	1.278945	1.58E−25	5.36E−24
IPO4	0.561749	1.389169	1.306225	7.09E−24	1.59E−22
NPM3	9.54458	24.74776	1.374545	1.97E−24	4.74E−23
RPP40	1.273059	3.302673	1.375335	1.34E−22	2.30E−21
GAPDH	294.9389	769.9426	1.384335	4.45E−22	6.93E−21
BRCA1	0.765346	2.059424	1.428057	1.31E−16	8.60E−16
BOP1	6.217107	16.99724	1.450985	1.62E−24	4.12E−23
NANOS1	0.335808	0.928977	1.468008	3.63E−14	1.82E−13
ZC3HAV1L	1.155211	3.320241	1.523131	1.18E−24	3.12E−23
ZNF239	0.874584	2.672671	1.611613	2.39E−25	7.21E−24
SNRPA1	0.015434	0.928897	5.911293	1.29E−09	3.59E−09
RPL3L	0.032343	0.121929	1.914499	8.50E−13	3.40E−12
ELAVL4	0.035481	0.140918	1.989722	6.76E−09	1.77E−08
PABPC1L	1.855937	7.506425	2.015978	4.80E−18	3.90E−17
DNMT3B	0.392316	1.608128	2.035293	4.80E−24	1.10E−22
SRSF12	0.129762	0.549804	2.083045	2.44E−16	1.58E−15
RPL39L	3.546697	15.04962	2.085179	3.96E−19	3.52E−18
TDRD12	0.049896	0.227115	2.186435	0.000647	0.000987
SRRM3	0.146264	0.723182	2.305785	5.10E−21	6.32E−20
EZH2	0.886476	4.871041	2.458076	1.65E−31	7.28E−29
RNASE10	0.022611	0.124294	2.458674	2.74E−07	5.98E−07
DAZL	0.00942	0.052944	2.490656	4.53E−06	8.59E−06
CALR3	0.0071	0.040493	2.511851	2.97E−06	5.75E−06
DDX4	0.005041	0.035423	2.813018	4.79E−10	1.42E−09
DQX1	0.059981	0.442106	2.881807	7.43E−11	2.36E−10
ERN2	0.633938	4.926838	2.958249	0.028185	0.035405
BOLL	0.00495	0.040354	3.027155	0.000102	0.000172
CELF5	0.028814	0.26448	3.198332	2.55E−13	1.12E−12
ELAVL2	0.021215	0.225389	3.40925	1.39E−05	2.54E−05
EXO1	0.215066	2.434663	3.500873	2.45E−30	4.06E−28
RDM1	0.042535	0.483658	3.50726	1.04E−27	5.30E−26
MAEL	0.100127	1.347756	3.750662	0.026688	0.033717
TDRD5	0.065196	0.912056	3.806273	7.80E−09	2.02E−08
IGF2BP3	0.120158	1.791874	3.89847	6.42E−13	2.61E−12
A1CF	0.005314	0.085191	4.002745	0.01586	0.02055
MEX3A	0.295238	5.148278	4.12414	6.98E−32	4.62E−29
RBM46	0.006302	0.126857	4.331187	0.000275	0.000437
YBX2	0.053701	1.347808	4.649513	6.94E−20	6.96E−19
CELF3	0.028839	0.798452	4.791134	0.000273	0.000435
PIWIL1	0.002602	0.075065	4.850517	3.81E−10	1.14E−09
TERT	0.005867	0.248854	5.406519	1.35E−28	9.95E−27
KHDC1L	0.014078	0.613746	5.446112	2.36E−07	5.22E−07
IGF2BP1	5.048866	8.458856	1.744503	1.10E−20	1.24E−19
EEF1A2	0.450645	34.93159	6.276398	8.75E−17	5.85E−16
LIN28A	0.004205	0.34273	6.348789	0.02661	0.03365
APOBEC1	0.003075	0.926701	8.235297	6.28E−10	1.83E−09

### 2.5 Cell culture and cell transfection

Human lung adenocarcinoma cell lines (A549 and H1299) and normal lung epithelial cell (Beas‐2B) were purchased from The Type Culture Collection of the Chinese Academy of Science, Shanghai, China. Cells were both maintained in DMEM/Ham's F‐12 (1:1) (DF‐12) medium supplied with 10% fetal bovine serum (Hyclone, Logan, HA) in a humidified atmosphere with 5% CO_2_ at 37°C.

The small‐short hairpins RNA against *SNRPA1*, along with negative control, were purchased from Gene Pharma (Shanghai, China) (Table S1). The transfection process was as described following the manufacture protocol.^13^

### 2.6 Immunohistochemistry and image analysis

Three‐micron‐thick sequential sections were obtained from FFPE tumor blocks of 56 LUAD patients and 70 normal lung tissues collected among 2010 to 2017. Microarray of samples was incubated in citric acid antigen retrieval buffer (pH 6.0) at 100°C, and intrinsic peroxidase activity was blocked by 3% hydrogen peroxide. Blocked with 1% normal goat serum, the slides were incubated with SNRPA1 primary antibody (1:100; ProteinTech, Wuhan, China) overnight. Following treatment with antirabbit secondary antibodies (ProteinTech), diaminobenzidine (DAB) chromogen substrate and hematoxylin were used to detect indicated molecular and nuclei staining, respectively.

### 2.7 Cell viability assays

For cell viability detection, approximately 500 cells were plated in six‐well plates for 10 days and fixed with 4% paraformaldehyde for 30 min. After being washed with PBS, cells were stained with crystal violet. A number of cells ≥50 were defined as one colony.

In the meantime, 10 000 cells transfected with siRNA were plated in 24‐well plates, then each well was incubated with 50‐μM BrdUrd medium for 2 h. After being fixed in 4% paraformaldehyde and permeated with PBS containing 0.3% Triton X‐100, cells were incubated with indicated staining solution (Beyotime, Shanghai, China) according to the manufacturer's protocol. Hoechst 3342 was performed to stain the cell nuclei, and positive cells were observed by fluorescence microscopy.

### 2.8 Cell wounding healing and transwell assay

To detect cell migration capacity, cells within full confluence were scratched and maintained with serum‐free medium for another 24 h. The scratches at 0 and 24 h were photographed, and the gap size of each group was calculated by Image J software (National Institute of Health, Bethesda, MD, USA).

Transwell chamber (BD Bioscience Pharmingen, San Jose, CA, USA) coated with Matrigel was employed to examine invasive abilities of LUAD cells. First, A549 and H1299 were transfected with siRNAs, and then mixed with serum‐free medium. Approximately 2 × 10^4^ cells were introduced into the upper chamber, and the lower chamber was placed with medium containing 10% FBS; 24 h later, noninvasive cells were removed, and the invasive cells were analyzed using a microscope.

### 2.9 Immunoblotting assays

The transfected cells were lysed with radio‐immunoprecipitation assay (Beyotime) supplied with proteinase and phosphatase inhibitor cocktails. After being incubated at 4°C for 30 min, the lysates were collected and denaturized. A total of 20‐μg protein was separated onto sodium dodecyl sulphate polyacrylamide gel electrophoresis (Epizyme, Shanghai, China) and transferred onto polyvinylidene fluoride membranes (Millipore, Billerica, MA, USA). Blocked with 5% non‐skimmed milk, the membranes were immunoblotted with primary antibodies against SNRPA1 (1:1000, cat. no. 17368‐1‐AP), N‐cadherin (1:1000, cat. no. 22018‐1‐AP), Vimentin (1:20000, cat. no. 10366‐1‐AP), TGF‐β Polyclonal antibody (1:2000, cat. no. 21898‐1‐AP), or β‐actin (1:50 000, cat. no. 66009‐1‐Ig) (all antibodies purchased from ProteinTech Inc., Wuhan, China) overnight, respectively. After 18 h of incubation, the membranes were washed with Tris‐buffered saline buffer containing 0.1% Tween‐20 and then incubated with HRP‐conjugated Affinipure Goat Anti‐Rabbit IgG (H + L) (1:8000, cat. no. SA00001‐2) or HRP‐conjugated Affinipure Goat Anti‐Mouse IgG (H + L) (1:10000, cat. no. SA00001‐1). Finally, the indicated blots were detected following enhanced chemiluminescence (Tanon, Shanghai, China) and analyzed with Image J software.

### 2.10 Statistical analysis

The data are presented as the mean ± SD. Data analysis was conducted using Graph Pad Prism 19.0 and analyzed using paired *t*‐test between two groups. One‐way ANOVA with Tukey's post hoc test was performed to compare more than two groups. Differences with *p* value <0.05 were regarded as statistically significant.

## 3 RESULTS

### 3.1 High expression of SNPRA1 leads to poor prognosis of LUAD

After strictly screenings using the R package, a total of 116 significantly differentially expressed hub‐RBPs (47 downregulated and 69 upregulated) was identified (Table 1). Then, significant differentially expressed RBPs were analyzed using the STRING database and ranked according to degree using Cystoscope (Table S2 & Figure S1). Through selection following the Kaplan–Meier method and univariate Cox regression methods, four candidate genes (*GAPDH*, *IGF2BP1*, *PABPC1L*, and *SNRPA1*) were identified, among which *SNRPA1* was the most significantly dysregulated RBPs associated with the overall survival time (OS) in LUAD (Table S3).

**FIGURE 1:**
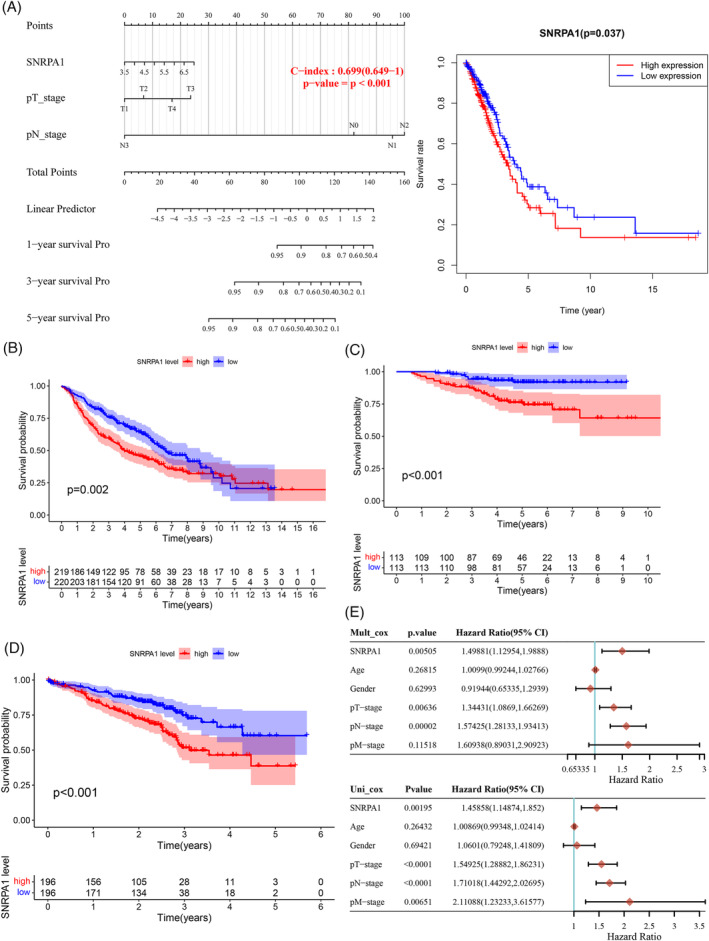
The survival curves of SNRPA1. Association between expression of SNRPA1 and overall survival in TCGA datasets (A); and the correlations were further verified in another GEO databases, including GSE68465 (*n* = 442), GSE31210 (*n* = 226), and GSE72094 (*n* = 442) (B–D). (E) Univariate and multivariate Cox regression of SNRPA1 expression and clinical stage characteristics of LUAD.

Consistently, Kaplan–Meier analysis and data from another three cohorts of LUAD patients (GSE68465, GSE31210, and GSE72094) both indicated that the OS of LUAD patients with high *SNPRA1* expression was shorter than the OS of patients with low *SNRPA1* expression. Next, in the univariate and multivariate analyses, high levels of SNRPA1 protein, as well as increased TN pathological stage, was the independent factor for a worse outcome of LUAD (Figure 1). All these results suggested that SNPRA1 was a reliable diagnostic index and a risk factor for LUAD.

### 3.2 Highly expressed SNRPA1 in LUAD patients and cell lines

**FIGURE 2:**
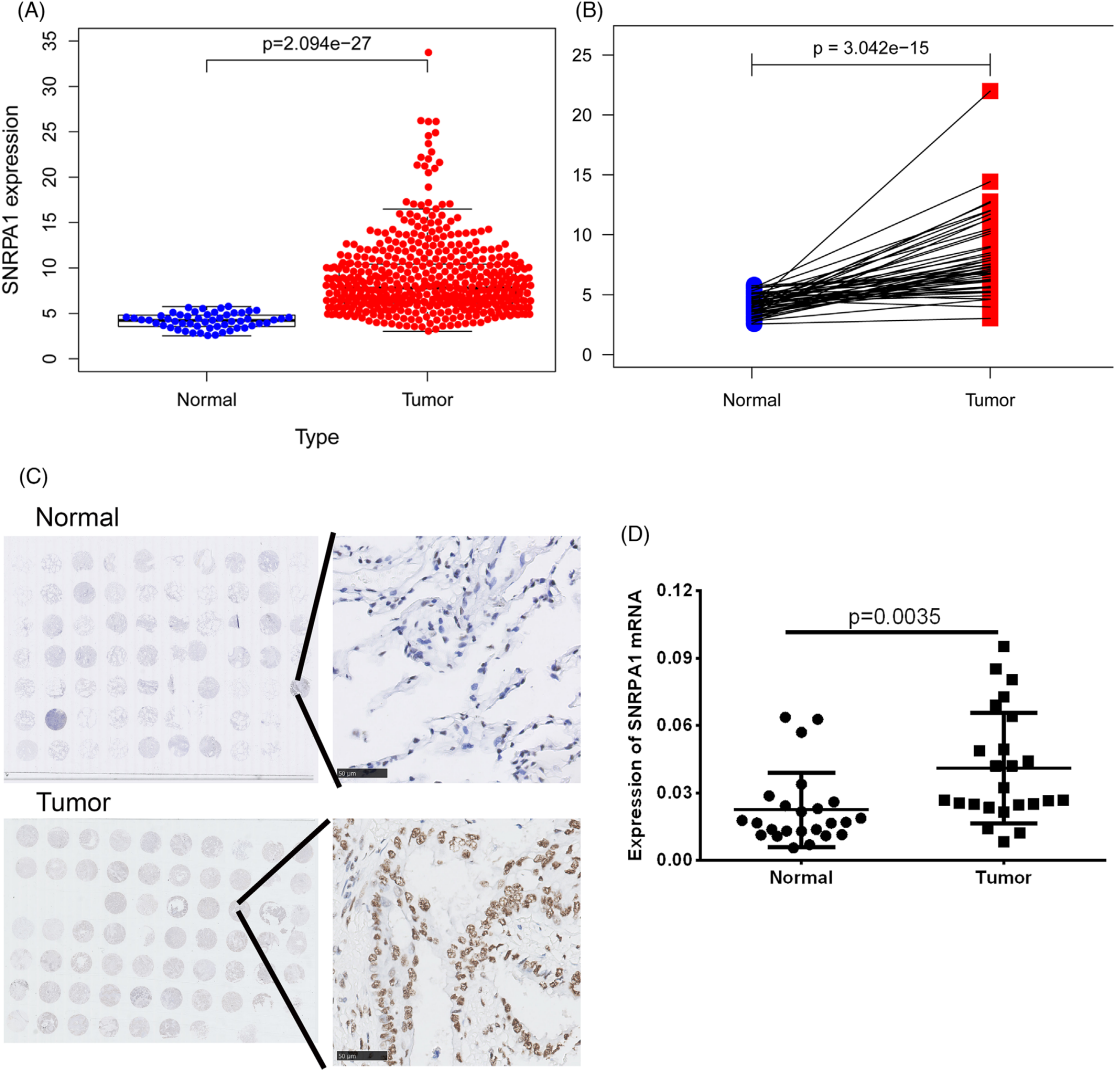
External validation of SNRPA1 in multiple databases and clinical specimens. (A,B) *SNRPA1* expression between LUAD and normal samples in studies of TCGA and GEPIA databases. (C) The immunohistochemistry results of SNRPA1 in 56 pairs LUAD tissues and 70 pan‐adjacent normal tissues. (D) The *SNRPA1* mRNA levels in paired LUAD patients (*n* = 16).

**FIGURE 3:**
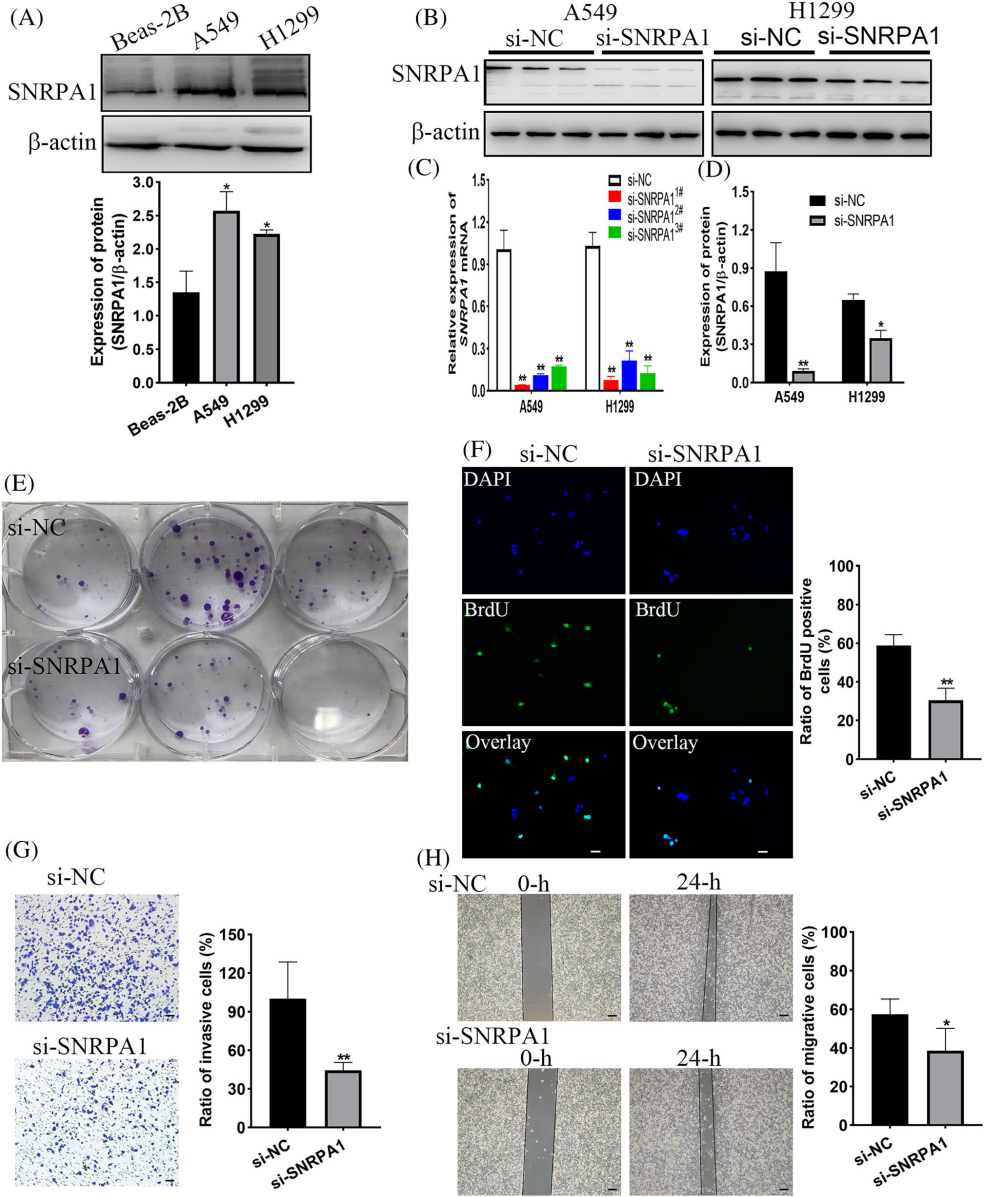
Validation the function of SNRPA1 on cell proliferation, invasion, and migration in A549 cell lines. (A) Expression of SNRPA1 in lung adenocarcinoma and normal lung epithelial cells. (B–D) mRNA levels of *SNRPA1* in A549 and H1299 cells after being transfected with NC and SNRPA1 siRNA. (E) Clone formation ability of A549 cell after transfected with si‐NC and si‐SNRPA1. (F) Proliferation capacity of A549 cells verified by BrdU corporation assay, scare bar, 50 μm. (G) Cell invasion of A549 cell after transfected with si‐NC and si‐SNRPA1. (H) Wound healing of A549 cells after transfected with si‐NC and si‐SNRPA1. Scare bar, 50 μm. **p < 0.01, compared with si‐NC groups. [Correction added on 25 July 2023, after first online publication: Figure 3 has been corrected.]

To ascertain the expression patterns of SNRPA1 in LUAD, TCGA and GEPIA online database first revealed the elevated level of SNRPA1 in tumor tissues (Figure 2A). Further, *SNRPA1* mRNA and protein levels are notably increased in tumor tissues compared with adjacent normal tissues (Figure 2B–D). In the meantime, as compared with normal lung epithelial cell line Beas‐2B, we observed the increased levels of SNRPA1 in LUAD cell lines, especially for A549 and H1299 cell lines (Figure 3A).

### 3.3 *SNRPA1* knockdown decreases the malignant behavior of LUAD cells

**FIGURE 4:**
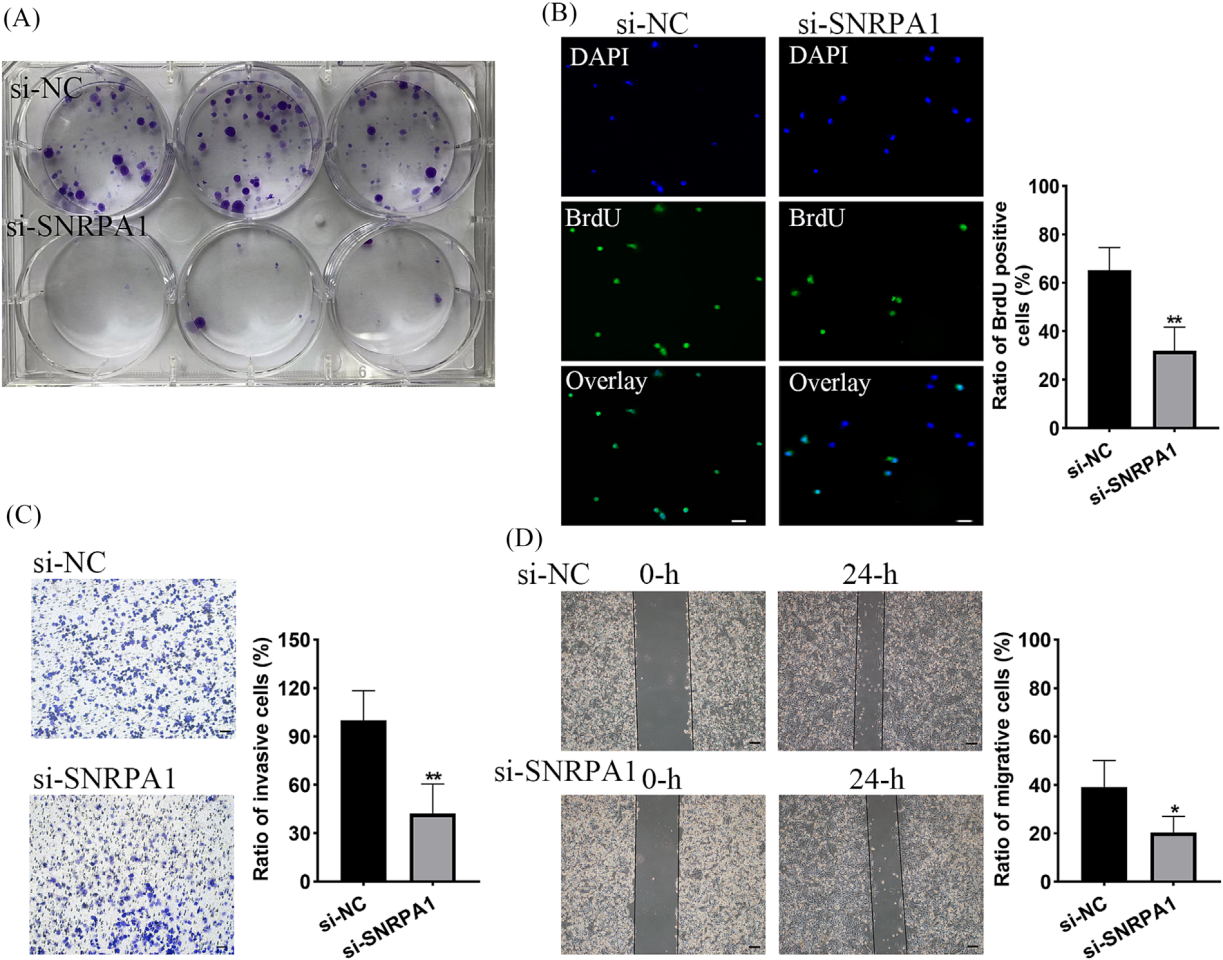
Validation the function of SNRPA1 on cell proliferation, invasion and migration in H1299 cell lines. (A) Clone formation ability of H1299 cell after transfected with si‐NC and si‐SNRPA1. (B) Proliferation capacity of H1299 cells verified by BrdU corporation assay, scare bar, 50 μm. (C) Cell invasion of H1299 cell after transfected with si‐NC and si‐SNRPA1. (D) Wound healing of H1299 cells after transfected with si‐NC and si‐SNRPA1. Scare bar, 50 μm. **p < 0.01, compared with si‐NC groups.

The prognosis value of SNRPA1 in LUAD patients prompted us to verify whether SNRPA1 might be involved in oncogene function. After silencing *SNRPA1*, mRNA levels of *SNRPA1* noticeably decreased in A549 and H1299 cells (Figure 3B). Colony formation assays and BrdU corporation detection showed that the proliferation rates of A549 cell was significantly inhibited with *SNRPA1* knockdown (Figure 3C–3D); hence, numbers of invasive cells and migrate rates obviously decreased in *SNRPA1* silenced A549 cells (Figure 3E,F). Consistently, *SNRPA1* silencing decreased the proliferation, invasion and migration rates of H1299 cells (Figure 4). Therefore, the target therapy to SNRPA1 might be a potential new strategy for malignancy of LUAD.

### 3.4 Identification of SNRPA1‐related signaling pathways

To explore the potential function of SNRPA1 in LUAD tumorigenesis, we conducted KEGG enrichment analysis to predict SNRPA1‐related signaling pathways. The results showed that high‐level of SNRPA1 was correlated with the activation of tumor proliferation signature, DNA repair, G2 checkpoint, and DNA replication, whereas low SNRPA1 substantially associated with inactivation of degradation of ECM, TGF‐β signaling pathway, the process of apoptosis and angiogenesis, as well as IL‐10 anti‐inflammatory signaling and inflammatory response (Figure S2).

**FIGURE 5:**
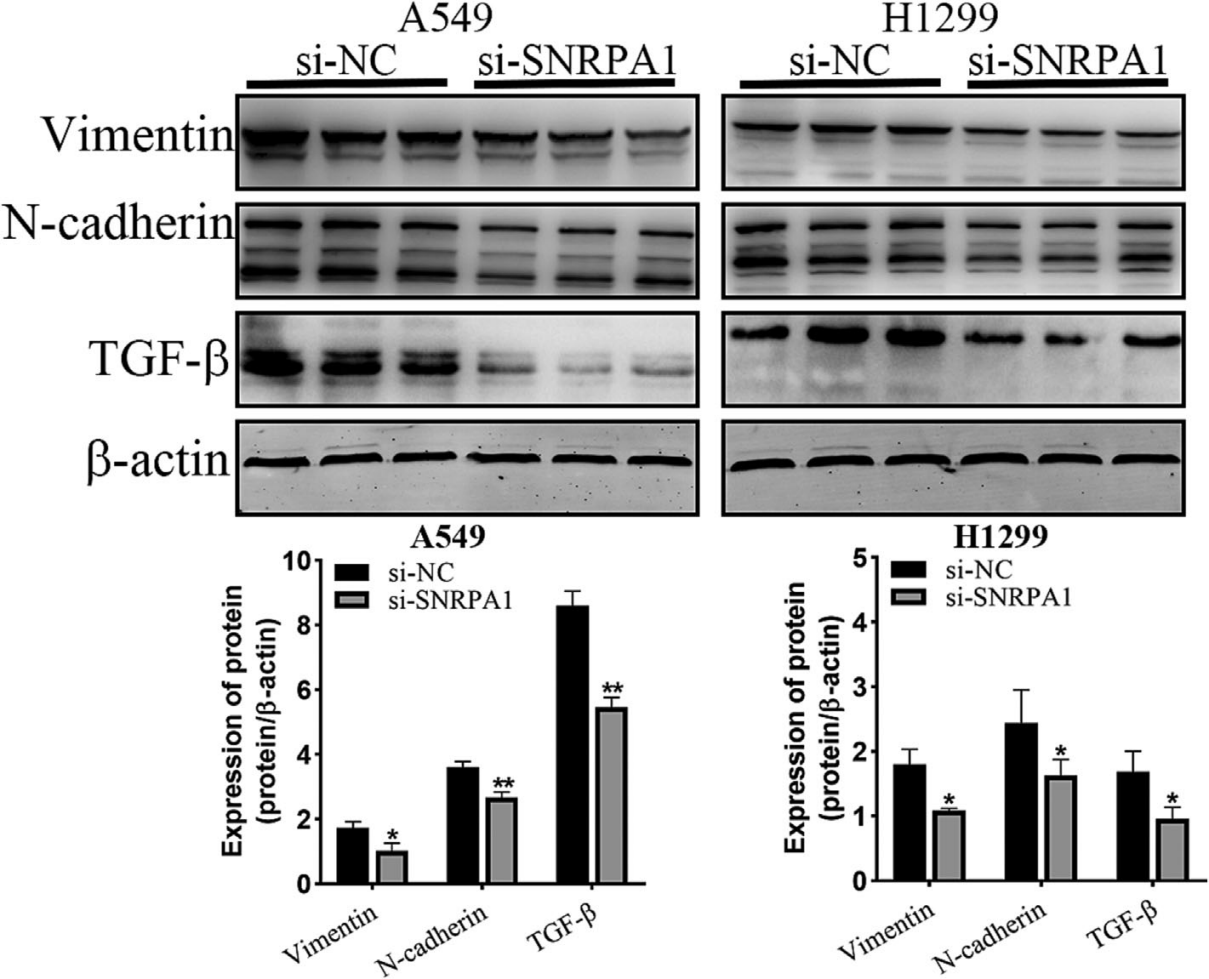
SNRPA1 knockdown inhibited EMT process and TGF‐β signaling pathway in A549 and H1299 cells. Protein levels of SNRPA1, Vimentin, N‐cadherin, and TGF‐β were identified by western blot assays, and the density of indicated band was analyzed using Image J software.

Among these enrichment pathways, transforming growth factor‐β (TGF‐β) functioned as a tumor promoter to promote tumor invasion and metastasis. As expected, we observed high expression of TGF‐β in A549 and H1299 cells, while knock down of SNRPA1 could notably decreased the expression of TGF‐β. Importantly, TGF‐β‐derived EMT is correlated with poor prognosis and lung metastasis. Herein, we found that *SNRPA1* silencing decreased the expression of mesenchymal markers, N‐cadherin and Vimentin both in A549 and H1299 cells (Figure 5). These data suggest that *SNRPA1* promotes the EMT process and TGF‐β signaling in lung adenocarcinoma.

### 3.5 Effect of SNRPA1 on the activation of immune microenvironment

**FIGURE 6:**
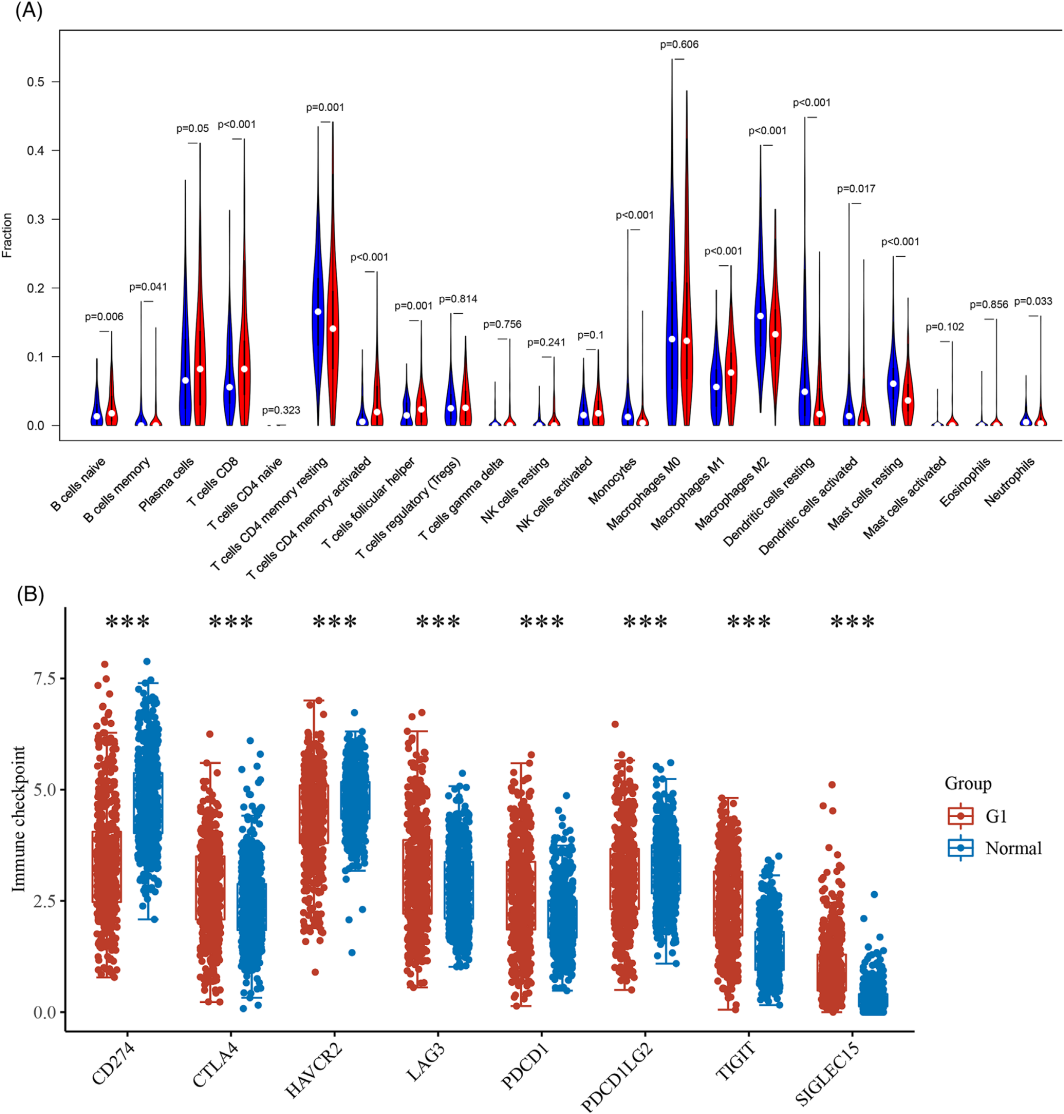
Correlation between SNRPA1 and immune microenvironment. (A) Correlation between SNRPA1 expression and infiltration scores of 28 immune cells in LUAD. (B) Correlation between immune checkpoints and SNRPA1 expression.

Considering the vital role of immune microenvironment in the progression of tumors, we evaluated the correlation between SNRPA1 expression and immunocyte infiltration in LUAD using the TIMER database. The data indicated that SNRPA1 significantly positively associated with the infiltration of B cells, CD4+ T cells, CD8+ T cells, neutrophil, macrophage, and dendritic cells (Figures 6A and S3). In addition, the evaluation between SNRPA1 and immune checkpoints also revealed that the SNRPA1 level was notably associated with the increased level of immune checkpoints in LUAD, especially for PDCD1, CD274, PDCD1LG2, CTLA4, and LAG3 (Figure 6B). Altogether, the above results indicated that SNRPA1 may be involved in the immune response in the tumor microenvironment, and LUAD patients with low SNRPA1 might benefit form anti‐PD‐L1 therapy.

## 4 DISCUSSION

Briefly, many studies have evaluated gene expression profiles in LUAD including miRNAs, long non‐coding RNAs (lncRNAs), and m6A‐related genes^14^, ^15^; however, the systematic evaluation of the role of RBPs in LUAD has not been elucidated. In the current study, according to the stepwise multivariate Cox regression for significantly dysregulated RBPs in LUAD, four candidate genes were regarded as prognosis‐related RBPs. Among these genes, *IGF2BP1* have been proven to be associated with metastasis in various types of human cancers^16^, ^17^; *PABPC1L* is not only related to prognosis of prostate cancer, also the depletion of which could inhibit the migration of human colorectal cancer cells^18^, ^19^; however, the role of SNRPA1 in LUAD has not been elucidated.

As small nuclear ribonucleoprotein polypeptide A, SNRPA1 was differentially expressed in most cancer types.^9^, ^20^ Similarly, the elevated level of SNRPA1 both in RNA‐seq datasets and the clinical specimens were confirmed in LUAD patients (Figure 2). Besides, upregulation of SNRPA1 was correlated with the clinical stage and worse prognosis of LUAD patients (Figure 1). We also revealed that SNRPA1 deletion notably inhibited the cell proliferation, migration, and invasion both in A549 and H1299 cells (Figures 3 and 4); these results are consistent with the findings in ccRCC and breast cancer.^9^, ^11^ Further mechanism study let us know that SNRPA1 impacted the tumorigenesis of LUAD through promotion of cell cycle, activation of PI3K/AKT/mTOR signaling pathway and epithelial–mesenchymal transition (EMT) differentiation, and inactivation of several signals (such as TGF‐β and p53 signaling pathway) (Figure S2).

Previous study indicated RBPs can modulate the alternative splicing, stability, and translation of mRNAs during EMT. For example, Hu‐antigen R binds to the transcription factor snail and stabilizes Snail mRNA, resulting in promoting the EMT of pancreatic cancer^21^ as well as the RNA binding motif single stranded interacting protein 3 (RBMS3) interacts with the mRNA of EMT transcription factor PRRX1, promoting mesenchymal phenotype transition and invasion of triple‐breast cancer.^22^ Moreover, growing evidence has suggested that several signaling pathways, including TGF‐β, Wnt/β‐catenin, have been characterized as potent inducers of EMT, and the alternative RNA splicing acts as a critical regulator of these signals.^23^ In this study, we found that *SNRPA1* silencing decreased the protein levels of some EMT markers and TGF‐β (Figure 5), while whether *SNRPA1* promoted EMT progression potentially through binding and stabilizing some transcription factor or negatively regulating TGF‐β signaling pathway needs to be verified in the future study.

To date, an increasing number of studies have found that the features of immunocyte infiltration were correlated with immunotherapeutic responsiveness in LUAD.^24^ Furthermore, it is reported that the expressions of CTLA4, PD‐1, LAG‐3, and PD‐L1 were correlated with immunosuppression of the tumor microenvironment.^25^, ^26^ The previous study indicated that CTLA4 was highly expressed and positively correlated with the clinical stage of non‐small cell lung cancer,^27^ and CTLA4 blockade could promote the infiltration of immune cells and derived loss of Treg stability in glycolysis‐low tumor.^28^ In the current study, we revealed the positive association between SNRPA1 and immunocyte infiltration, as well as the immune checkpoints, especially CTLA‐4, PD‐L1, and LAG‐3 (Figure 6). Therefore, the combination blockade of SNRPA1 with immunotherapies might obtain synergistic antitumor activity.

## 5 CONCLUSIONS

For the first time, we recognized the prognostic value of SNRPA1 in LUAD by bioinformatic analysis and in vitro experiments. Collectively, not only elevated SNRPA1 could act as the independent risky prognostic factor, the blockade of SNRPA1 provided synergistic antitumor activity for immunotherapy.

## AUTHOR CONTRIBUTIONS

**Juan Juan Yang**: Conceptualization; methodology; writing—original draft; visualization. **Yu Jia Yang**: Writing—original draft; visualization; bioinformatics analyses. **Yi Lu Gu**: Data curation; methodology. **Li Tong**: Data curation; bioinformatics analyses. **Yi Fei Liu:** Conceptualization; supervision; funding support. **Jian Guo Zhang:** Investigation; validation.

## CONFLICT OF INTEREST STATEMENT

The authors declare that they have no competing interests.

## INFORMED CONSENT STATEMENT

Informed consent was obtained from all subjects involved in the study.

## ETHICS STATEMENT

All microarray data were downloaded from the databases. The study was conducted according to the guidelines of the Declaration of Helsinki, and approved by the Ethics Committee of Affiliated Hospital of Nantong University (protocol code 2021‐K133 and approved on 1st Jan, 2021).

## Supporting information


**Figure S1** Gene module based on cytocubba for candidate hub RBPs. Red and green represents up‐regulated and down‐regulated hub RBPs.
Figure S2 KEGG term enrichment analyses of SNRPA1 in LUAD.
Figure S3 Correlation between SNRPA1 and immune cell infiltration in LUAD.
**Table S1** Sequences for small‐short hairpins RNA and qRT‐PCR
**Table S2** Results of Log‐Rank test. HR: hazard ratio.
**Table S3** Results of univariate and multivariate Cox regression analyses between RBPs and DFS. RBPs: RNA‐binding proteins; HR: hazard ratio; DFS: disease‐free survival.

## Data Availability

The data that support the findings of this study are available on request from the corresponding author. The data are not publicly available due to privacy or ethical restrictions.
