# Supplementary material for: High SNRPA1 expression leads to poor prognosis in patients with lung adenocarcinoma
Source: Clin Respir J. 2023 Jun 5;17(8):719–32. doi: 10.1111/crj.13647 (PMC10435942; doi:10.1111/crj.13647)
Supplement: Supplementary file 1 — Figure S1 Gene module based on cytocubba for candidate hub RBPs. Red and green represents up‐regulated and down‐regulated hub RBPs. Figure S2 KEGG term enrichment analyses of SNRPA1 in LUAD. Figure S3 Correlation between SNRPA1 and immune cell infiltration in LUAD. Table S1 Sequences for small‐short hairpins RNA and qRT‐PCR Table S2 Results of Log‐Rank test. HR: hazard ratio. Table S3 Results of univariate and multivariate Cox regression analyses between RBPs and DFS. RBPs: RNA‐binding proteins; HR: hazard ratio; DFS: disease‐free survival. [file CRJ-17-719-s001.pdf]

Figure S1

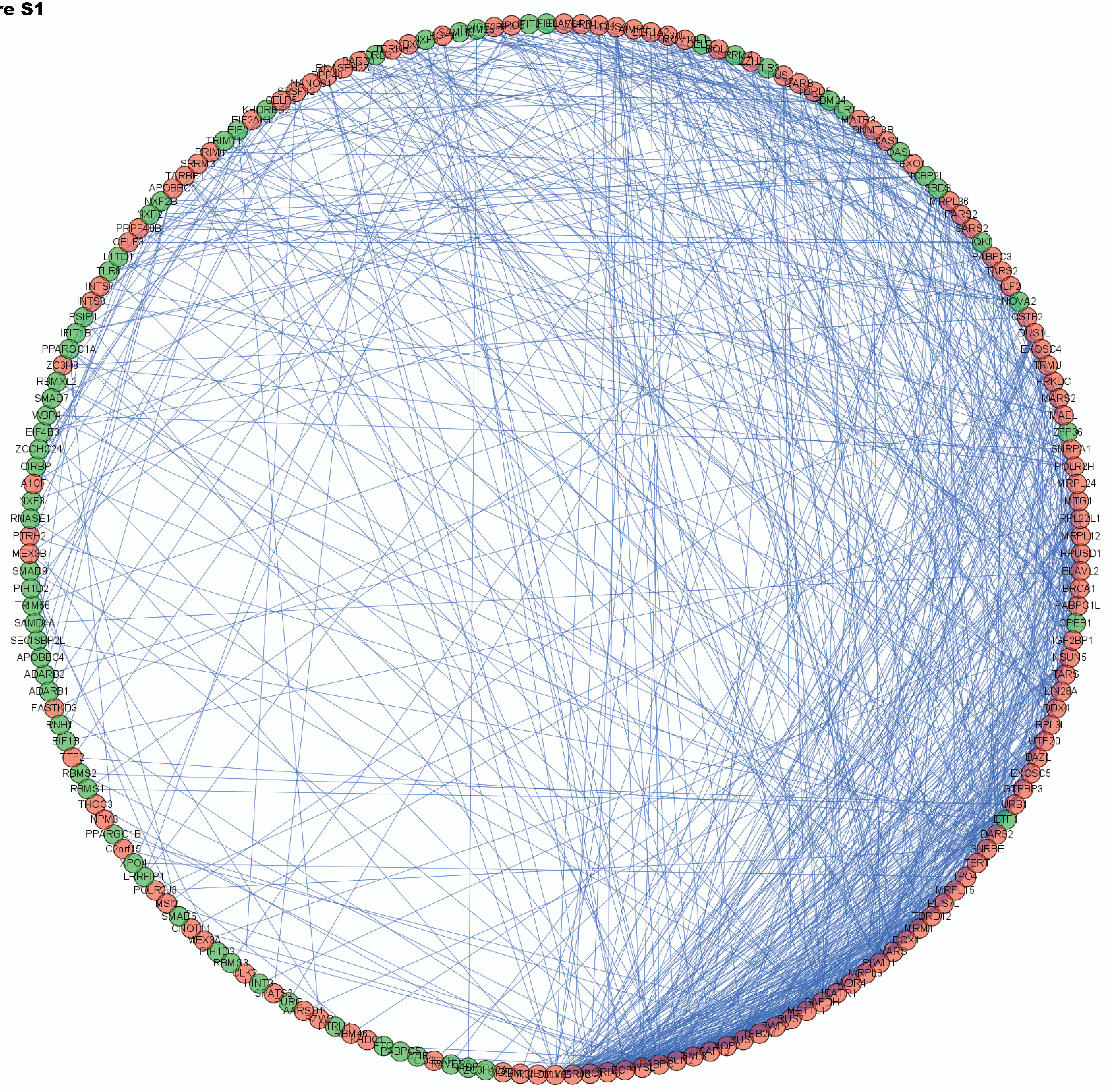

SRRM3
TARBP1
APOBEC1
NXF2B
NXF1
PRPF40B
CELF3
L1TD1
TLR8
INTS7
INTS8
PSIP1
IFIT1B
PPARGC1A
ZC3H8
RBMXL2
SMAD7
WBP4
EIF4E3
ZCCHC24
CIRBP
A1CF
NXF3
RNASE1
PTRH2
MEX3B
SMAD9
PIH1D2
TRIM56
SAMD4A
SECISBP2L
APOBEC4
ADARB2
ADARB1
FASTKD3
RNH1
EIF1B
TTF2
RBMS2
RBMS1
THOC3
NPM3
PPARGC1B
C2orf15
XPO4
LRRFIP1
POLR2J3
MSI2
SMAD6
MEX3A
PIH1D3
RBMS3
HINT3
AARSD1
RBM46
FTO
MATR3
DNMT3B
RBM24
NCBP2L
SBDS
MRPL36
PARS2
SARS2
QKI
PABPC3
TARS2
ILF2
NOVA2
CSTF2
DUS1L
EXOSC4
TRMU
PRKDC
MARS2
MAEL
ZFP36
SNRPA1
POLR2H
MRPL24
MTG1
RPL22L1
MRPL12
RPUSD1
ELAVL2
BRCA1
PABPC1L
CPEB1
IGF2BP1
NSUN5
TARS
LIN28A
DDX4
RPL3L
UTP20
DAZL
EXOSC5
GTPBP3
URB1
DARS2
SNRPE
TERT
MRPL15
TDRD12
MRM1
PIWIL1
HEATR1
GAPDH
METTL1

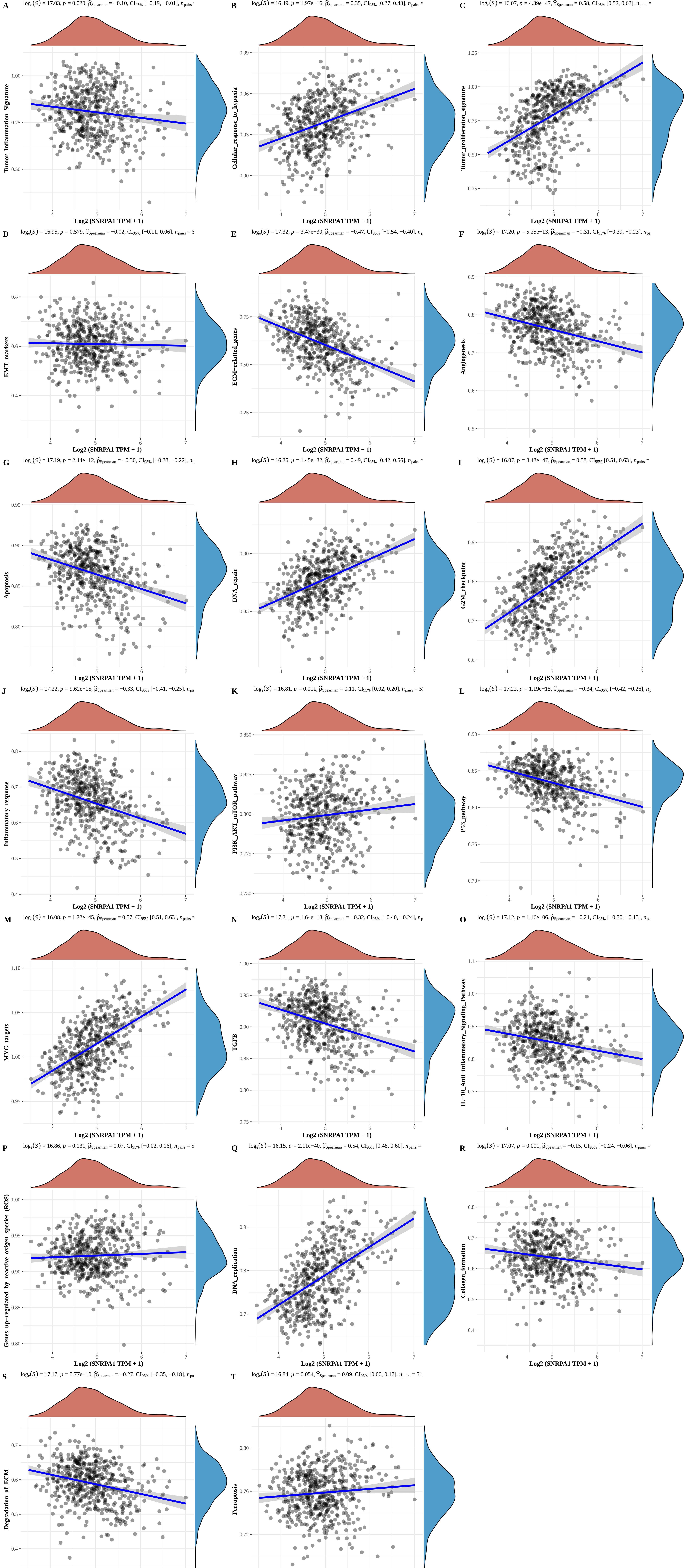
A
Tumor_Inflammation_Signature
B
Cellular_response_to_hypoxia
C
Tumor_proliferation_signature
D
EMT_markers
E
ECM−related_genes
F
Angiogenesis
G
Apoptosis
H
DNA_repair
I
G2M_checkpoint
J
Inflammatory_response
K
PI3K_AKT_mTOR_pathway
L
P53_pathway
M
MYC_targets
N
TGFB
O
IL−10_Anti−inflammatory_Signaling_Pathway
P
Genes_up−regulated_by_reactive_oxigen_species_(ROS)
Q
DNA_replication
R
Collagen_formation
S
Degradation_of_ECM
T
Ferroptosis
Log2 (SNRPA1 TPM + 1)

Figure S2

Figure S3

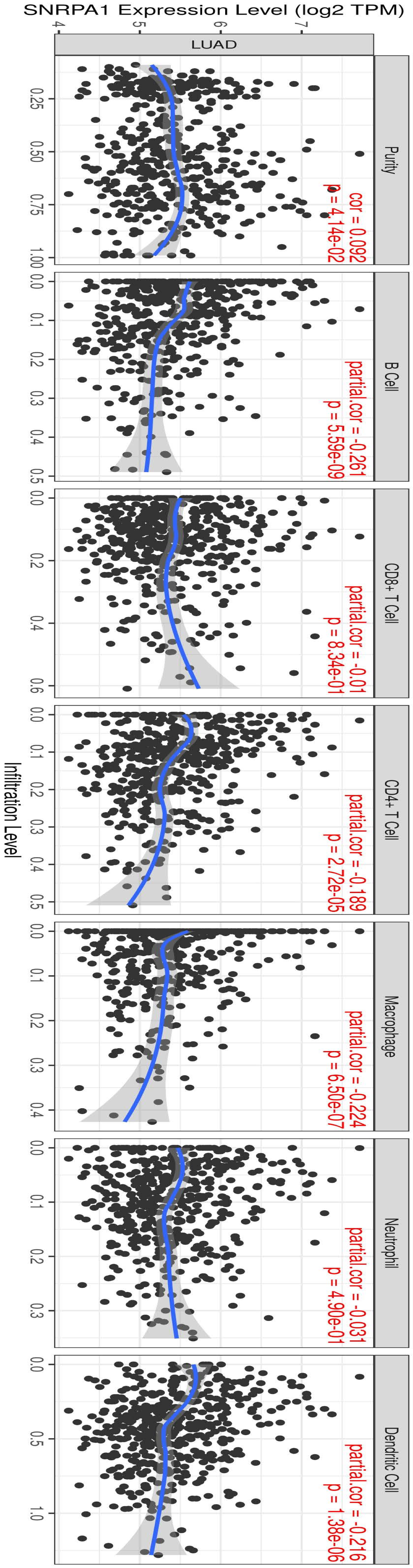

SNRPA1 Expression Level (log2 TPM)
LUAD
Infiltration Level
Purity
cor = 0.092
p = 4.14e-02
B Cell
partial.cor = -0.261
p = 5.59e-09
CD8+ T Cell
partial.cor = -0.01
p = 8.34e-01
CD4+ T Cell
partial.cor = -0.189
p = 2.72e-05
Macrophage
partial.cor = -0.224
p = 6.50e-07
Neutrophil
partial.cor = -0.031
p = 4.90e-01
Dendritic Cell
partial.cor = -0.216
p = 1.38e-06

Table S1. Sequences for small-short hairpins RNA and qRT-PCR

Gene		Sequence (5′ to 3′)
SNRAP1-siRNA1#	sense	5′-CCGUAUAGGUGAGGGACUUTT-3′
	anti-sense	5′-AAGUCCCUCACCUACCUAUACGGTT-3′
SNRAP1-siRNA2#	sense	5′-GGUAACCAAUAAGAAGCAUTT-3′
	anti-sense	5′-AUGCUUCUUAUUGGUUACCTT-3′
SNRAP1-siRNA3#	sense	5′-UCAGAUCCCUGGCAGAGAATT-3′
	anti-sense	5′-UUCUCUGCCAGGGAUCUGATT-3′
si-NC	sense	5′-UUCUCCGAACGUGUCACGUTT-3′
	anti-sense	5′-ACGUGACACGUUCGGAGAATT-3′
SNRPA1_PCR	Forward	5'-ATGCCGTATAGGTGAGGGAC-3'
	Reverse	5'-ATCACCCAGTTCCACGAGAC-3'
β-actin_PCR	Forward	5'-AGTTGCGTTACACCCTTTCTTG-3'
	Reverse	5'-GCTGTCACCTTCACCGTTCC-3'

Table S2. Log-Rank test results

Gene	HR	HR.95L	HR.95H	p-value
GAPDH	1.00061923	1.00018607	1.00105259	0.00507684
IGF2BP1	1.05510936	1.0114632	1.10063891	0.01281901
PABPC1L	0.97527449	0.95160954	0.99952794	0.04575585
SNRPA1	1.08508547	1.04663371	1.12494989	9.17E-06

Table S3. Univariate Cox regression Analysis

Gene	HR	HR.95L	HR.95H	p-value
GAPDH	1.00065927	1.0002579	1.00106079	0.00128298
SNRPA1	1.07395486	1.037621	1.111561	4.84E-05
IGF2BP1	1.04377146	1.0136012	1.07483975	0.00420046
PABPC1L	0.9725027	0.94874688	0.99685335	0.02712357
